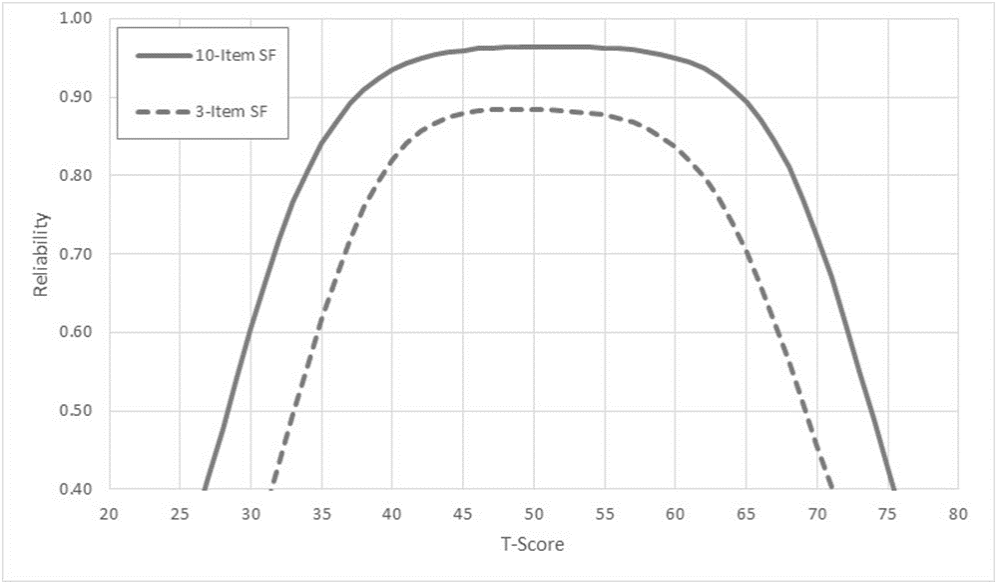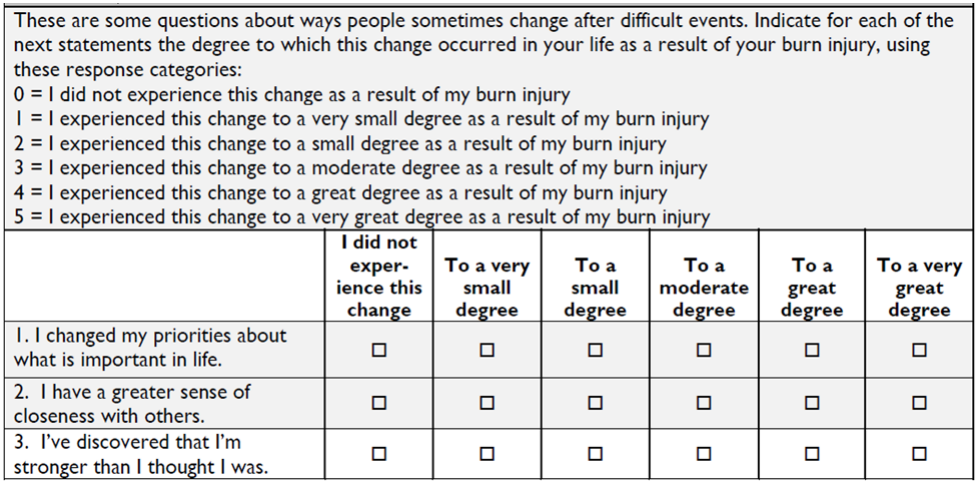# 5 New Short Form of the Posttraumatic Growth Inventory for Use in Individuals with Burn Injury

**DOI:** 10.1093/jbcr/iraf019.005

**Published:** 2025-04-01

**Authors:** Alyssa Bamer, Kara McMullen, Andrew Humbert, Kimberly Roaten, Jeffrey Schneider, Shelley Wiechman, Dagmar Amtmann

**Affiliations:** University of Washington; University of Washington; University of Washington; University of Texas Southwestern; Spaulding Rehabilitation Hospital, Harvard Medical School; Harborview Medical Center, University of Washington; University of Washington

## Abstract

**Introduction:**

The Posttraumatic Growth Inventory (PTGI) was developed to measure the positive psychological changes that individuals can experience after trauma. The original scale contained 21 items measuring three categories of perceived benefits based on posttraumatic growth theory: changes in self-perception, interpersonal relationships, and philosophy of life. Prior research identified five data derived subfactors, which were used to create a 10-item short form (two items each). While the 10-item form is relatively brief, an even shorter version was desired to reduce participant response burden. Thus, the aim of this study was to create a briefer version of the PTGI using item response theory (IRT) that can detect group differences (i.e. reliability >0.8) and is suitable for use in individuals with burn injury.

**Methods:**

The 10-item PTGI was administered to 1,076 adults recovering from moderate to severe burn injury between 6-months and 20-years after injury as part of an ongoing longitudinal study. Items were fit to Samejima’s graded response model if unidimensionality was supported by a 1-factor confirmatory factor analysis (CFA). Local dependance (LD) was examined using the X2 statistic in IRTPRO software. A focus group of burn clinicians, data collectors, and psychometricians selected items for a new short form while considering item content, acceptability, and reliability.

**Results:**

CFA confirmed unidimensionality, but LD results indicated that items were highly redundant. A new three item short form was created and includes one item from each of the three categories of perceived benefits identified in posttraumatic growth theory. Reliability of the new short form is moderate (>0.8) for scores ±1 SD around the mean (figure 1). Raw summary scores on the new 3-item short form correlate highly (r=0.94) with summary scores on the 10-item short form.

**Conclusions:**

The new 3-item short form (figure 2) of the PTGI has reliability sufficient for group comparisons and summary scores correlate highly with the 10-item version. The new short form balances item content and items are acceptable to people with burn injury.

**Applicability of Research to Practice:**

This short form represents less burden to patients and can be used to assess posttraumatic growth in a clinic or research setting.

**Funding for the Study:**

N/A